# Effectiveness of creative arts therapies/expressive arts therapy for psychosocial outcomes in adults with oncological disease: an umbrella review protocol

**DOI:** 10.3389/fpsyg.2025.1570798

**Published:** 2025-08-08

**Authors:** Magdalena Svozilová, Jirí Kantor, Zuzana Svobodová, Alžběta Smrčková, Miloslav Klugar

**Affiliations:** ^1^Center of Evidence-Based Education and Arts Therapies: A JBI Affiliated Group, Faculty of Education, Palacky University Olomouc, Olomouc, Czechia; ^2^Faculty of Education, Institute of Special Education Studies, Palacky University Olomouc, Olomouc, Czechia; ^3^Faculty of Health Sciences, Palacky University Olomouc, Olomouc, Czechia; ^4^Cochrane Czech Republic, Czech Republic: a JBI Centre of Excellence, Czech GRADE Network, Institute of Health Information and Statistics of the Czech Republic, Prague, Czechia

**Keywords:** creative arts therapies, oncology, protocol, umbrella review, psychosocial outcomes

## Abstract

This umbrella review will evaluate the effectiveness of creative arts therapies (CAT)—encompassing art, drama, dance/movement, and music therapies—compared to standard non-creative treatments, in enhancing psychosocial outcomes for adults with oncological diseases. Using the JBI methodology for umbrella reviews, we will conduct systematic searches across MEDLINE, Open Dissertations, ProQuest Central, Epistemonikos, and PsycINFO databases, without restrictions on publication year or language. Eligible studies will include systematic reviews of quantitative evidence assessing CAT interventions' effects on psychosocial outcomes, such as depression, anxiety, and quality of life. Screening of studies, qualitative appraisal, data extraction, and data synthesis will be conducted by two independent reviewers. The quality of evidence will be evaluated using the GRADE approach. This protocol is registered with PROSPERO (ID: CRD42023410121).

## 1 Introduction

Despite numerous studies on creative arts therapies (CAT), many existing systematic reviews lack consistency in terms of population characteristics, intervention types, and outcome measurement tools, which hinders a comprehensive synthesis of their effects. As a result, practitioners and policy makers lack a reliable source of information to guide their practice and decision making. This umbrella review aims to fill this gap by systematically synthesizing the findings of existing systematic reviews across all major CAT modalities and outcome domains, thereby providing an integrative and methodologically rigorous overview currently lacking in the literature.The study will synthesize evidence from existing systematic reviews to provide a comprehensive overview of the effectiveness of CAT, allowing for a high-level comparison of the efficacy of different therapeutic approaches and highlighting areas of strong or weak evidence.

Cancer is considered one of the most common causes of death. 19.3 million new cancer cases and almost 10.0 million cancer deaths occurred in 2020 (Sung et al., 2021). A cancer diagnosis may result in many physical and psychosocial problems. Cancer patients experience severe negative symptoms related to diagnosis, treatment, and disease progression. The main symptoms are a decreasing quality of life, pain, fatigue, depression, and anxiety. Mood disorders occur in 30% to 40% of patients (Mitchell et al., 2011). One of the inseparable factors is the adverse effect of stress on the body, significantly reducing the quality of life (Kerr et al., 2016). Treatment primarily focusing on tumor reduction has several side effects: impairment of many bodily functions (including sexuality), complications in working life, spirituality and deterioration of one's financial status (Lewandowska et al., 2020). Somatic and psychological symptoms often overlap, and it is difficult for an expert to say whether a sleep disorder, decreased appetite, or pain is caused by the side effects of treatment or the patient's psychological experience (DeVita et al., 2018). Treating a person with cancer is exceptionally challenging, and patients need support in their psychosocial health. The range and frequency of interventions focused on patients' mental health is also increasing due to decreased costs for other medical services (Rieger et al., 2021).

With a close relationship to psycho-oncology and integrative medicine, CAT belong to a group of interventions currently used for cancer treatment (The British Association of Art Therapists, 2023). “Creative arts therapies” is an umbrella term for healthcare professions that use arts—creative and expressive processes—to improve and enhance the psychological and social wellbeing of individuals of all ages and health conditions (Shafir et al., 2020). CAT offer supportive ways to deal with the negative experiences of life with oncology disease. These therapies are alternative and complementary treatments focused mainly on psychosocial outcomes. Besides many commonalities, there are also some essential differences between concerning the therapeutic practice and the current state of the research (Kerr et al., 2016). The main disciplines are art therapy, dance/movement therapy, drama therapy, and music therapy.

CAT represent a collective term for therapeutic approaches that use artistic and expressive processes—such as art therapy, dance/movement therapy, drama therapy, and music therapy—to improve an individual's psychological and social wellbeing. These therapies focus on promoting emotional expression, personal growth, and enhanced quality of life through active engagement in creative artistic activities.

To ensure conceptual clarity, this review distinguishes between creative arts therapies (CAT), expressive arts therapy (EAT), and several related but excluded approaches. Although these interventions all incorporate artistic modalities, they differ fundamentally in their purpose, structure, and professional standards.

Creative arts therapies (CAT) refer to regulated health professions—such as art therapy, music therapy, dance/movement therapy, and drama therapy—that employ specific art forms within a psychotherapeutic framework. These therapies are delivered by professionally trained practitioners, often with formal certification or licensure, and are grounded in psychological theory. A structured therapeutic relationship is central to the process, and the use of the artistic modality is intentional, goal-directed, and clinically focused.

Expressive arts therapy (EAT) is a distinct integrative approach characterized by the multimodal use of two or more art forms within a single therapeutic process. It emphasizes the creative process itself—rather than any one artistic medium—as the primary means of self-expression, emotional integration, and healing. While EAT is also facilitated within a therapeutic relationship, it tends to prioritize flexibility, improvisation, and symbolic exploration. Regulation of EAT practice varies across countries and contexts.

By contrast, related practices that are excluded from this review—such as music medicine, vibroacoustic therapy, or general therapeutic arts—often lack a formal therapeutic relationship, do not require specialized training in psychotherapy, and may focus on passive reception (e.g., listening to music) or general wellbeing rather than specific psychosocial outcomes. Although these approaches may offer benefits in healthcare or supportive environments, they fall outside the scope of this umbrella review, which focuses exclusively on professionally facilitated CAT and EAT interventions with defined therapeutic intent.

Expressive Arts Therapies refer to a specific approach that is based on the multimodal use of various art forms—including visual art, music, dance, drama, writing, or poetry—and emphasizes the integration of these modalities within the therapeutic process. The aim is to provide participants with an environment where they can process their emotions, cope with challenging life situations, and build psychological resilience through artistic expression.

Art therapy (AT) is a form of psychotherapy that uses art media as its primary mode of communication. The overall aim of its practitioners is to enable a client to change and grow on a personal level, using art materials in a safe and facilitating environment (The British Association of Art Therapists, 2023). Artmaking may positively affect anxiety, depression, and QoL in adults with cancer. Most of the 654 patients included in a study by Bosman considered an experience of art therapy beneficial to their wellbeing (Bosman et al., 2021).

Studies focused on AT and self-image in patients with breast cancer present that approaching emotions through time-limited art therapy treatment seems to have a long-lasting effect on the attachment behavioral system shown in the Structural Analysis of Social Behavior model post intervention, and this effect remained 5 years later. There were significant improvements in three SABS clusters: Autonomous self, Accepting self, and Loving self (Thyme et al., 2022). Participants of one session with AT intervention before chemotherapy demonstrated a decrease in symptoms of anxiety, drowsiness, and tiredness (De Feudis et al., 2021). The patient can learn coping strategies, accept the fact of living with cancer, and find feelings of belonging in the safe setting of the group.

Dance/movement therapy (DMT) is mainly researched in women with breast cancer. Quality of life may be improved after DMT intervention, and fatigue, depression, stress, and anxiety can be decreased (Fatkulina et al., 2021). In the embodied approach, patients can learn to connect sensory and affective cues with cognition, verbalization, and behavior. Due to the transition between emotions, body, and cognition, new patterns can be generalized into everyday life (Koch and Fischman, 2011). The investigation showed that DMT is beneficial in reducing the side effects of radiation therapy, such as pain, stress, anxiety, and fear, giving a psychotherapeutic relief (Vardhan et al., 2022). Participation in DMT sessions can also promote the sharing of feelings, help decrease feelings of isolation and loneliness, and support feelings of solidarity and togetherness (Boing et al., 2018). DMT was found to be an effective intervention in the treatment of adults with depression (Karkou et al., 2019).

Drama therapy (DT) is the intentional use of drama and/or theater processes to achieve therapeutic goals. DT is an embodied practice that is active and experiential. This approach can provide the context for participants to tell their stories, set goals and solve problems, express feelings, or achieve catharsis (Berghs et al., 2022). Drama therapy intervention research is relatively scarce compared to other CAT modalities. DT could deliver significantly positive effects and improve the self-awareness, self-expression, interpersonal interaction and communication skills, self-recognition ability, social role ability, and decision-making ability of participants (Chang et al., 2019). The results of a recent systematic review summarize the different approaches used in DT and the populations with which it is being used. In the last decade, DT research has produced promising results, showing that drama therapy offers effective treatment for various populations (Orkibi and Feniger-Schaal, 2019).

Music therapy (MT) is the clinical and evidence-based use of music interventions to accomplish individualized goals within a therapeutic relationship by a credentialed professional who has completed an approved music therapy program (American Music Therapy Association, 2005). Music therapy interventions can address a variety of healthcare and educational goals (Stegemann et al., 2019). It can positively impact life on behavioral, cognitive, emotional, perceptual, and spiritual levels (Edwards, 2015). Music therapy can help promote patients' emotional regulation, adaptation, coping, and increase their sense of control (Daykin et al., 2007).

Expressive arts therapy is a specific approach based on a multimodal usage of various modalities - visual art, music, dance/movement, drama (including both, DT and psychodrama), play, and poetry/writing (Daykin et al., 2007). Besides CAT and expressive arts therapy, there are other related disciplines using arts for therapeutic purposes, e.g., music medicine, therapeutic arts, or vibroacoustic therapy (Stegemann et al., 2019; Kantor et al., 2022). Although CAT and related disciplines often overlap regarding the usage of techniques, therapeutic goals, procedures, or the focus on artistic experience, professionals from CAT differ in providing a strong emphasis on the development of a therapeutic relationship through artistic experience, and attaining proper education/professional certification in any of the CAT disciplines (Bucharová et al., 2020). The expected amount of knowledge and skills required is higher for CAT practitioners than for other healthcare professionals. As a result, the effectiveness of CAT disciplines may be different compared to related approaches in supporting psychosocial outcomes in patients with oncologic disease. Therefore, this umbrella review will focus on all CAT modalities and expressive arts therapy but will not consider the related approaches. Moreover, there is a need to support comparative research that would help to identify the commonalities and differences, as well as the unique contributions of all these CAT disciplines (Kantor et al., 2021). Oncologic disease is a suitable population for this purpose, as there is a high number of research studies in some CAT disciplines. This is true primarily for music therapy, where a relatively high number of studies titled as systematic reviews was found (see Appendix A). Aside from conflicting results found in some studies, there is a potential risk of research waste. It is useful to explore the state of evidence synthesis in this area of healthcare practice and provide rigorous guidance that would inform CAT practitioners, policymakers, and researchers about the potential of CAT disciplines in the treatment of adult people with oncologic disease. Furthermore, researchers in these disciplines may benefit from recommendations on how to improve the status and quality of research. All these issues may be addressed by a study with an umbrella review design. Because a preliminary search of PROSPERO, MEDLINE, the Cochrane Database of Systematic Reviews and the JBI Evidence Synthesis found no current or registered umbrella review on the topic, we prospectively registered (ID:CRD42023410121) and published this protocol of a planned umbrella review.

### 1.1 Review question

What is the effectiveness of creative arts therapies/expressive arts therapy on psychosocial outcomes in adult patients with oncological diseases?

## 2 Methods and analysis

### 2.1 Study design

This review will follow the JBI methodology for umbrella reviews (Aromataris et al., 2024) and will adhere to the PRISMA guidelines to ensure transparent reporting (Page et al., 2021).

### 2.2 Search strategy

This review will apply a three-step search strategy. In the first step, the relevant key words from abstracts/titles and the index terms to describe the papers were find through an initial search of MEDLINE (accessed through PubMed) and Epistemonikos. Based on these findings, search strategies for different databases were developed (see Appendix B for a search strategy for Epistemonikos). In the second step, a comprehensive literature search will be conducted across multiple electronic databases, including:

- MEDLINE (via PubMed)
- Web of Science
- ProQuest Central
- PsycINFO
- Epistemonikos

Gray literature sources (Open Dissertations) will also be included, to search for dissertations and master theses; however, bachelor theses will be excluded. Any publication form will be eligible—journal article, conference proceedings, thesis, book, or chapter in a book. As a third step, the reference lists of all included studies will be checked for additional studies. There will be no restrictions on publication year or language.

In accordance with the JBI methodology for umbrella reviews (Aromataris et al., 2024), outcome-related terms (e.g., “anxiety,” “depression,” “quality of life”) were intentionally not included in the search strategy. This decision was made to ensure maximum sensitivity and to avoid excluding relevant systematic reviews that report psychosocial outcomes without listing them in the title or abstract.

Psychosocial outcomes are clearly defined in Section 2.3.4 of this protocol and will be identified during the screening and data extraction phases. This approach is particularly suitable for umbrella reviews, where outcomes may be broad and heterogeneously reported across the literature.

As recommended by the JBI Manual for Evidence Synthesis, a broad search strategy based on population and intervention terms supports a comprehensive identification of relevant evidence, while outcome selection is refined during the review process.

### 2.3 Eligibility criteria

#### 2.3.1 Participants

This umbrella review will consider studies that include adult oncological patients (18 years and older) who have experienced or are diagnosed with cancer or have recovered from any oncological disease. For systematic reviews including both children and adults, only data pertaining to adults will be extracted. Patients in any stage of disease or treatment will be included and with any type of malignancy. Participants' gender, demographic, economic, and sociocultural context will not be limited.

#### 2.3.2 Interventions

This review will consider studies that evaluate the impact of any CAT discipline (art therapy, dance/movement therapy, drama therapy, and music therapy) or expressive arts therapy.

Given the global heterogeneity in credentialing standards for CAT professionals, we will not restrict inclusion to interventions delivered by legally certified therapists. However, information on the professional background and training of facilitators will be extracted where available, as differences in practitioner qualifications may contribute to methodological and clinical heterogeneity.

Interventions must be explicitly described by the authors as creative arts therapy (CAT) or expressive arts therapy and must, by their nature, reflect the essential characteristics of these approaches. This includes the use of artistic modalities as a core component of the therapeutic process, the presence of a therapeutic relationship, and a psychotherapeutic or psychosocial goal. Interventions that lack these core attributes, or that rely solely on passive exposure to the arts without structured therapeutic intent, will be excluded.

For the purposes of this protocol, creative arts therapies and expressive arts therapies are not treated as separate entities in the search strategy. We will include both categories in our search for systematic reviews, ensuring an inclusive strategy that captures all relevant literature. The inclusion and exclusion criteria are the same for both groups.

Once the studies have been identified, we will subsequently categorize and sort them based on the specific modalities and types of interventions as described in the original studies. This categorization will allow us to provide a more detailed comparison of the effectiveness of the different approaches while ensuring that the methodological criteria for inclusion remain consistent throughout the umbrella review.

We will not include interventions of related disciplines, such as music medicine, drama education, self-care intervention, used by the patient (without clearly described therapeutic relationship). Interventions that use artistic modalities without aesthetic purposes will be excluded. If the study evaluates the effectiveness of different interventions, we will extract data solely for CAT if possible.

#### 2.3.3 Comparators

This review will consider studies that compare the CAT interventions with standard treatment, verbal psychotherapy, or other complementary treatment which does not contain creative arts therapies. Standard complementary treatment refers to widely accepted interventions commonly offered in hospitals, such as mind-body techniques (e.g., relaxation exercises), nutritional counseling, physical activity programs, psychosocial support services (e.g., counseling), pain management strategies, and symptom management.

#### 2.3.4 Outcomes

This review will consider studies that include psychosocial outcomes. As primary outcomes, we will consider:

- Depression measured, e.g., by the Patient Health Questionnaire-9 (PHQ-9).
- Anxiety measured, e.g., by the State-Trait Anxiety Inventory (STAI).
- Distress measured, e.g., by Distress Thermometer (DT).
- Social and spiritual support measured, e.g., by Multidimensional Scale of Perceived Social Support (MSPSS).

Secondary outcomes are expected to be quality of life, loneliness, communication, and body image. However, we will consider any other outcome that adheres to the psychosocial area of a patient's functioning.

#### 2.3.5 Types of studies

Only systematic reviews from studies exploring the effectiveness of CAT/expressive arts therapy treatment in adult patients with oncologic disease will be eligible for inclusion in this umbrella review. We will consider systematic reviews with or without meta-analysis, as well as systematic reviews including various designs of quantitative research (not only randomized controlled trials). Excluded will be all types of non-systematic reviews and overview studies such as literature/narrative reviews, scoping reviews, critical reviews, or integrative reviews.

### 2.4 Data screening

Following the search, all identified citations will be collated and uploaded into Rayyan and duplicates removed. Following a pilot screening of two independent reviewers, titles and abstracts will then be screened by two independent reviewers for assessment against the inclusion criteria for the review. Potentially relevant papers will be retrieved in full text. The full text of selected citations will be assessed in detail against the inclusion criteria by two independent reviewers. Authors of the included studies will be contacted in the case of missing information needed for inclusion of the potentially relevant studies or data synthesis in the following stage. Reasons for exclusion of full text articles that do not meet the inclusion criteria will be recorded and reported in the umbrella review. Any disagreements that arise between the reviewers at each stage of the selection process will be resolved through discussion, or with a third reviewer. The results of the search will be reported in full in the final report and presented in a Preferred Reporting Items for Systematic Reviews and Meta-analyses (PRISMA) flow diagram (Page et al., 2021).

### 2.5 Critical appraisal of included studies/assessment of methodological quality

The selected studies will be critically appraised by two independent reviewers for methodological quality using the standardized critical appraisal instrument from JBI. The appraisal process will be piloted by the reviewers. Where necessary, authors of papers will be contacted to request missing or additional data for clarification. Any disagreements between the reviewers will be resolved through discussion, or with a third reviewer.

The eleven criteria on the JBI checklist will be assessed and assigned a result (i.e., yes/no/other specific response) applicable to each criterion. The classification of each review into high, moderate, low, or very low quality will be determined based on the number of “no” responses: fewer than two “no” responses will indicate high quality, two to three “no” responses will indicate moderate quality, four to five “no” responses will indicate low quality, and six or more “no” responses will indicate very low quality. Additionally, certain criteria, such as a “no” response to question number three, will automatically classify a study as low quality.

The results of the JBI appraisal will be reported in both narrative form and a tabulated summary. Regardless of methodological quality, all studies will undergo data extraction and synthesis. The results of the quality assessments will be used to help contextualize the evidence base of the umbrella review and discuss to what extent the quality of the included systematic reviews may have affected the comprehensiveness and results of the umbrella review.

### 2.6 Data extraction and management

Data will be extracted from the systematic reviews included in this umbrella review by two independent reviewers. Any discrepancies will be resolved through discussion or, if necessary, consultation with a third reviewer.

An example of the data extraction table has been developed and piloted during the preparation of this protocol. A summary table of study characteristics is provided in Appendix D.

The extracted data will include:

- Study characteristics (author; year; title; type of review; number of included primary studies; instrument used for assessing the risk of bias).
- Population details (number of participants; their age; gender; type of cancer; demographic characteristics). Demographic information including age, gender, ethnicity, and cultural background will be extracted where available to allow for interpretation of results in light of intersectional factors.
- Intervention characteristics (CAT modality; specific forms, methods, or approaches used; length and frequency of the intervention).
- Comparator used (including length and frequency of treatment).
- Outcomes assessed [type of outcome, e.g., anxiety, depression, quality of life; self-reported/clinician-rated; rates and methods of analysis; meta-analysis details; results of certainty of findings assessment (see the table in Appendix C)].

### 2.7 Study matching

We will perform study matching to evaluate the number of included primary studies in each systematic review and its influence on the results of review. This will be reported in tabular form.

### 2.8 Data synthesis

The above data extracted from selected reviews will be tabulated and accompanied by narrative synthesis to address the review question. Findings may be reported according to:

- Specific modalities such as art therapy, music therapy, drama therapy and dance-movement therapy, and expressive arts therapies.
- Different comparators, namely CAT vs. standard treatment, CAT vs. verbal psychotherapy, and CAT vs. other complementary treatment which does not contain creative arts therapies.
- Type of outcomes—depression, anxiety, distress, social and spiritual support, and any other secondary outcomes. In the case of depression and anxiety, findings will be further classified as patient-reported outcomes, or clinically reported outcomes.
- Where sufficient data are available, subgroup analyses will be conducted to examine whether the effectiveness of CAT interventions varies by cancer type (e.g., breast cancer) and by treatment phase (e.g., active treatment, survivorship, or palliative care). This stratification will help contextualize the effects within relevant clinical subpopulations and enhance the interpretability of the results for practice.
- From the perspective of timing of follow-up measurement, studies will be divided according to the outcomes measured immediately after treatment (post-test measurement), up to half a year after treatment, and after half a year.

The findings will be summarized according to data related to the number of studies that inform the outcome, the date range, the number of participants (from included studies), and the heterogeneity of the results of the included systematic reviews. Confidence intervals, exact *p*-values (if provided), and effect sizes (risk/odds/hazard ratio, or standardized weighted difference) will be presented for systematic reviews with meta-analysis. The influence of the quality of included reviews on the results will be explored by sensitivity analyses, where studies of lower quality will be analyzed separately from those of moderate and high quality. The methodological and clinical heterogeneity of included reviews will be also reported. Any overlap among the studies will be reported and described in a tabular**/**graphical arrangement. The results of the umbrella review will be provided in tabular form in a “Summary of Evidence” table.

### 2.9 Assessing certainty of evidence

To assess the certainty of the evidence in the findings of this umbrella review, we will utilize the Grading of Recommendations, Assessment, Development and Evaluation (GRADE) approach where available. Specifically, when included systematic reviews have applied the GRADE methodology, these assessments will be adopted and reported in our umbrella review. Summary of Findings (SoF) tables will be created using GRADE (McMaster University, ON, Canada). These tables will include details about the population, intervention, comparator, and specific measures of each outcome, along with corresponding results (Stegemann et al., 2019). In cases where GRADE is not applied in the systematic reviews, a clear notation of this limitation will be included, and the level of evidence will be reported based on the available information without re-evaluating it using GRADE.

## 3 Results and discussion

This umbrella review will synthesize findings from existing systematic reviews on the effectiveness of CAT for improving psychosocial outcomes in adults with oncological diseases. Where possible, findings will be interpreted with attention to equity and inclusion, acknowledging that psychosocial responses to CAT may vary based on intersecting identity factors such as gender, culture, or socioeconomic status.

One key contribution of this umbrella review will be to explore the unique contribution of different CAT modalities and clarify the effectiveness of various CAT in specific psychosocial domains, such as anxiety, depression, and quality of life. This synthesis will address the existing fragmentation in the evidence base, providing a comprehensive understanding of which CAT modalities/types of intervention are most beneficial and for whom. This summary can serve as a valuable resource for healthcare providers, patients, and policymakers. The findings from this review are expected to have important implications for clinical practice by offering a robust evidence base to inform treatment decisions in oncology settings. Additionally, they may guide future research by highlighting areas where evidence is lacking or where further investigation is needed to support creative arts therapies in cancer care.

Potential limitations include variability in the quality and methodologies of the included systematic reviews and their primary studies, which could affect the overall conclusions. Additionally, publication bias may limit the availability of data from less successful interventions. Efforts will be made to address these challenges through careful quality assessment using the GRADE.

## 4 Ethics and dissemination

### 4.1 Ethics

This umbrella review will involve the analysis of secondary data from published systematic reviews, which does not require formal ethical approval. The data extracted will be publicly available, and no new primary data collection will take place.

### 4.2 Dissemination

The findings of this umbrella review will be disseminated through multiple channels to maximize impact. These channels include:

- Peer-reviewed publication: The review findings will be submitted for publication in a high-impact journal relevant to psycho-oncology and complementary therapies.
- Conference presentations: Results will be presented at both national and international conferences related to oncology and creative arts therapies.

Additionally, a summary of the findings will be made available through institutional and social media channels to ensure that patients and practitioners can easily access the results.
