# Supplementary material for: Effectiveness of creative arts therapies/expressive arts therapy for psychosocial outcomes in adults with oncological disease: an umbrella review protocol
Source: Front Psychol. 2025 Aug 8;16:1570798. doi: 10.3389/fpsyg.2025.1570798 (PMC12371950; doi:10.3389/fpsyg.2025.1570798)
Supplement: Supplementary file 1 [file Table_1.docx]

Example of Data Extraction Table

| **Author, Year** | Köhler et al., 2020 |
| --- | --- |
| **CAT Modality** | Music therapy |
| **Description of Intervention** | Active (singing, instrument playing) and receptive (listening) music therapy provided by trained therapists. |
| **Intervention Duration/Frequency** | Varied (1–20 sessions, 30–60 minutes/session) |
| **Comparator Type** | Standard care, wait-list, active control group |
| **Primary Outcomes** | Psychological well-being |
| **Measurement Tools** | HADS, STAI, EORTC QLQ-C30, BDI-II |
| **Meta-analytic Effect Sizes** | 0.35 (95% CI: 0.19–0.50), p < 0.001 |
| **95% Confidence Intervals** | 0.35 (95% CI: 0.19–0.50), p < 0.001 |
| **Heterogeneity (I²)** | I² = 36.39%, Q = 27.77, p = 0.066 |
| **Publication bias** | Egger’s test not significant; funnel plot asymmetry observed |
| **Analysis Method** | Random-effects model |
| **Certainty of Evidence (GRADE)** | Not reported |
| **Quality Appraisal Tool** | Cochrane Risk of Bias Tool |
| **Key Findings** | Significant moderate effects for anxiety and depression |
| **Notes** |  |
